# P02.137. Mindfulness-based stretching and deep breathing exercise reduces symptoms of posttraumatic stress disorder

**DOI:** 10.1186/1472-6882-12-S1-P193

**Published:** 2012-06-12

**Authors:** S Kim, M Burge

**Affiliations:** 1 Dept. of Health, Exercise & Sports Sciences, University of New Mexico, Santa Fe, USA; 2 Internal Medicine, School of Medicine, University of New Mexico, Albuquerque, USA

## Purpose

Nurses are known to experience high levels of work-related stress. The purpose of this study is to explore the relationships between changes in biomarkers (ACTH, DHEAS and cortisol) and posttraumatic stress disorder (PTSD) symptom severity resulting from nurses with high levels of stress participating in an 8-week program of mindfulness-based stretching and breathing exercise.

## Methods

Thirty-three nurses recruited from the University of New Mexico hospital underwent an 8-week exercise intervention for this randomized, crossover clinical trial. Participants were screened with the PTSD Checklist Civilian version (PCL-C). Participants were randomly assigned to either the wait-list control group or the exercise group. Cortisol, DHEAS, ACTH and PTSD symptom severity were measured at weeks 0, 4, 8, 12, and 16.

## Results

The study is currently underway. The preliminary data show that there is a negative relationship between changes in cortisol concentrations and PTSD symptom severity, as well as a positive relationship between changes in cortisol and ACTH levels, and between the ratio of cortisol/DHEA and symptom severity.

## Conclusion

The preliminary results provide a better understanding of the relationship between changes in PTSD symptom severity and biomarker levels as a result of the mindfulness-based intervention. This contribution is likely to be significant because it will advance knowledge about the physiological regulation anomalies of the autonomic nervous system associated with PTSD and increase our understanding of how mindfulness-based exercise affects PTSD symptomology.

